# Community water fluoridation policy and population oral health: a policy analysis

**DOI:** 10.3389/froh.2026.1847759

**Published:** 2026-07-16

**Authors:** Josefine Ortiz Wolfe, Matt Jacob, Susan Fisher-Owens

**Affiliations:** 1 College of Natural Sciences, The University of Texas at Austin, Austin, TX, United States; 2 Jacob Strategies, Arlington, VA, United States; 3 University of California San Francisco, San Francisco, CA, United States

**Keywords:** community water fluoridation, dental caries prevention, oral health equity, oral health policy analysis, population oral health, public health policy analysis

## Abstract

Dental caries (tooth decay) remains the most common noncommunicable disease worldwide and continues to impose significant dental and systemic health and economic burdens despite being largely preventable. Untreated disease contributes to pain, infection, reduced quality of life, and increased demand for restorative dental care. Governments, therefore, face policy decisions regarding population-level prevention strategies. This analysis evaluates policy options related to population-level fluoride exposure, including maintaining existing community water fluoridation (CWF) programs, expanding fluoridation coverage, discontinuing fluoridation in favor of clinical prevention strategies, and implementing alternative delivery mechanisms such as salt fluoridation. Drawing on Bardach's Eightfold Path for policy analysis, options are assessed using public health policy criteria, including health impact, equity implications, economic considerations, safety, and implementation feasibility. Evidence shows that CWF can reduce caries prevalence and treatment needs at the population level, particularly in communities with limited access to preventive dental care. Economic evaluations indicate that fluoridation reduces expenditures associated with dental treatment. However, policy feasibility varies depending on infrastructure, governance systems, and public health capacity. For policymakers, fluoridation represents one component of broader health prevention strategies that combine population-level prevention with improved access to preventive services.

## Introduction

Caries remains the most common noncommunicable disease worldwide and continues to pose a significant public health challenge despite being largely preventable (1). Global estimates indicate that untreated caries in permanent teeth affects approximately two billion people worldwide, while more than 500 million children experience caries in primary (“baby”) teeth. Worldwide, nearly 3 in 10 people have untreated caries in their permanent (“adult”) teeth (2). Untreated disease contributes to pain, infection, impaired nutrition, reduced quality of life, and missed days of school and work (3).

Although effective preventive measures exist, many health systems continue to rely heavily on treatment-oriented models of dental care (1). In many countries, oral health services are financed largely through out-of-pocket payments (4), limiting access to preventive services and allowing preventable disease to progress until treatment becomes urgent and costly. As a result, governments increasingly face policy questions about how to strengthen upstream prevention strategies that can reduce disease burden at the population level (5).

Fluoride reduces caries by strengthening weakened teeth and slowing the disease's progression. Community water fluoridation (CWF) has been implemented in many jurisdictions as a public health intervention designed to reduce dental caries by maintaining controlled levels of fluoride in public drinking water systems (6, 7). At the same time, CWF remains contested in some contexts, reflecting broader debates about public health authority, political decision-making, and public acceptance that extend beyond scientific evidence (8). Thus, governments face policy choices regarding whether to maintain, expand, modify, or replace existing fluoridation programs as part of broader oral health prevention strategies.

This policy analysis evaluates options available to governments seeking to reduce population-level caries through strategies that increase fluoride exposure. Drawing on Bardach's Eightfold Path for policy analysis (9), policy alternatives are assessed using commonly applied public health criteria, including health impact, equity implications, economic considerations, safety, and implementation feasibility.

Although this analysis draws on international evidence and comparative policy approaches, it is primarily intended to inform policymakers in higher-income settings with centralized water infrastructure while also considering alternative fluoride delivery strategies used in other global contexts.

### The policy problem: how should governments reduce the population burden of dental caries?

Caries is the most common noncommunicable disease globally (1) despite being largely preventable. For policymakers, the challenge is to decide how governments can implement prevention strategies that reduce the disease burden across entire populations. Untreated dental disease contributes to pain, infection, impaired nutrition, reduced quality of life, and missed days of school and work. These outcomes generate avoidable health expenditures and productivity losses, placing pressure on health systems and public budgets (10).

Social and structural conditions strongly shape the distribution of caries. Disease burden is consistently higher among populations with lower income, limited access to dental care, and weaker health system infrastructure (1). Rural communities, marginalized populations, and individuals without regular access to preventive services experience higher rates of untreated disease. In many countries, oral health systems remain heavily treatment-oriented and are financed largely through out-of-pocket payments (1). These structural features limit access to preventive care, allowing disease to progress until treatment becomes urgent and costly.

For government decision-makers, the response to pediatric caries disease burden presents a particularly contentious policy concern. Severe early childhood caries frequently requires intravenous antibiotics and treatment under general anesthesia in hospital settings, placing additional pressure on public hospitals and publicly financed insurance programs (11). Dental conditions also contribute to emergency department visits, which often address acute pain or infection, burdening the emergency department without resolving the underlying disease process (12). These patterns highlight gaps in upstream prevention.

Caries results in substantial economic costs. Global estimates suggest that oral diseases generate hundreds of billions of dollars annually in direct treatment costs and productivity losses (10), not to mention increased disease and costs from systemic diseases such as diabetes, stroke, and dementia. In these circumstances, policymakers must determine which prevention strategies can reduce disease burden while limiting avoidable public expenditures. For policymakers responsible for public budgets and workforce productivity, these costs underscore the importance of effective prevention strategies.

These conditions create a structural policy problem. Clinical care alone cannot address population-wide patterns of disease, and individual behavior change strategies do not fully address the environmental and social factors that influence oral health outcomes. Given the limited nature of public funding, policymakers must consider how population-level prevention strategies can be used to reduce disease risk across entire communities (13). Governments consider different fluoride modalities when determining how best to reduce the burden of caries and its sequelae.

## Governance and policy context

Decisions regarding drinking water quality are typically governed through national public health or environmental regulatory frameworks. National governments commonly establish drinking water standards and public health guidance. Implementation is carried out by regional authorities, municipal utilities, or public water operators responsible for managing water infrastructure. International organizations such as the World Health Organization provide guidance on drinking water safety and fluoride exposure. Yet decisions regarding fluoridation policies ultimately occur within national or subnational governance systems (14).

Countries vary in how they implement population-level exposure to fluoride as part of oral health prevention strategies. Some jurisdictions operate CWF programs that adjust fluoride concentrations in public water systems to reduce caries. Other countries rely primarily on alternative delivery mechanisms such as salt fluoridation, milk fluoridation, or widespread use of fluoride rinses, varnish or toothpaste (15, 16). Salt fluoridation programs have been implemented in many countries, particularly in Latin America and parts of Europe, often coordinated through national food regulation and nutrition policies.

Governance structures also influence how fluoridation policies are implemented within countries. Because water infrastructure is frequently managed at the municipal or regional level, fluoridation coverage may vary across jurisdictions. Urban areas served by centralized water systems are generally better positioned to implement CWF. In contrast, rural communities with decentralized water supplies or private wells may face greater implementation challenges (3).

In recent years, fluoridation policies have been revisited in multiple jurisdictions (17). Policy debates often reflect broader questions about public health authority, environmental exposure, public trust, and community autonomy (18). Differences in governance systems, regulatory authority, and the public's confidence in health institutions influence how governments evaluate fluoridation policies and determine which prevention strategies are feasible within their regulatory and infrastructure contexts.

## Policy options

Governments seeking to reduce the burden of caries can pursue several policy options related to population-level fluoride exposure. These approaches differ in their potential health impact, implementation requirements, and alignment with national public health priorities. For policymakers, the central question is how governments should support fluoride delivery strategies as part of broader oral health prevention systems.

### Option 1: maintain existing CWF programs

In jurisdictions where fluoridation infrastructure is already established, governments may choose to maintain current programs at recommended fluoride concentrations. This approach preserves an existing population-level prevention strategy that provides consistent fluoride exposure through public drinking water systems at the lowest per-person cost.

### Option 2: expand CWF coverage

Governments may also consider expanding fluoridation to additional populations served by centralized water systems where fluoridation has not yet been implemented. Expansion strategies typically involve installing fluoridation equipment, establishing monitoring systems, and integrating oversight into existing drinking water regulatory frameworks, with lower per-person costs but higher initial infrastructure costs.

### Option 3: discontinue water fluoridation and rely on clinical prevention strategies

Another option is to discontinue CWF and rely primarily on clinical and consumer-based prevention strategies. These approaches include fluoride toothpaste use, professional topical fluoride applications, dental sealants, and other preventive dental services delivered through health care systems. This decentralizes responsibility and cost.

### Option 4: implement alternative population-level fluoride delivery strategies

Some countries have pursued alternative approaches such as salt fluoridation delivered through regulated food production systems. Implementing these strategies typically requires coordination with food producers, monitoring of fluoride concentrations in commercial products, and national surveillance systems to track population exposure.

These options represent distinct policy pathways for governments seeking to reduce caries through population-level fluoride exposure strategies. The policy options presented in this analysis were synthesized from fluoridation approaches currently implemented or debated internationally, as well as from strategies described in the public health and oral health literature. The options were adapted to support comparative analysis within the policy context under review and include:

- maintaining existing CWF programs;
- expanding fluoridation coverage;
- discontinuing fluoridation in favor of clinical prevention strategies;
- and considering alternative population-level fluoride-delivery mechanisms, such as salt fluoridation.

The following sections evaluate these alternatives using public health policy criteria, including health impact, equity impact, economic implications, safety, and implementation feasibility.

## Evaluation framework

This policy analysis uses Bardach's Eightfold Path framework to systematically evaluate policy alternatives related to population-level fluoride exposure. The analysis operationalizes the framework by defining the policy challenge, assembling evidence, constructing policy alternatives, selecting evaluation criteria, projecting likely outcomes, confronting trade-offs, informing policy recommendations, and communicating the analysis in a structured narrative format. In this policy brief, these steps are applied through a comparative assessment of policy options rather than a formal quantitative scoring model. The analysis draws on a targeted review of systematic reviews, governmental and public health reports, and jurisdictional case studies relevant to fluoride policy. These sources informed the evaluation of public health effects, implementation considerations, and policy trade-offs associated with each option. More than 250 sources were reviewed, with priority given to recent systematic reviews, governmental evidence syntheses, and comparative policy analyses.

Evidence sources were identified through targeted searches of PubMed, Google Scholar, government public health websites, and reports from international health organizations. Search concepts included community water fluoridation, dental caries prevention, oral health equity, fluoride safety, economic evaluation, and fluoridation policy. Sources were evaluated for relevance to population-level fluoride interventions, oral health outcomes, policy implementation, economic considerations, and health equity. Sources with limited relevance to the policy questions under consideration were generally not incorporated into the final analysis. Additional details regarding evidence identification, selection, and prioritization are provided in Table 1.

**Table 1:** Evidence identification and selection process.

**Component**	**Description**
Evidence Sources	PubMed, Google Scholar, governmental public health websites, international health organization reports, and policy/evidence synthesis documents
Search Concepts	Community water fluoridation, dental caries prevention, oral health equity, fluoride safety, economic evaluation, oral health policy, population oral health, fluoridation policy, and policy analysis
Evidence Review	More than 250 evidence sources reviewed
Relevance Assessment	Sources evaluated for relevance to population oral health outcomes, policy implementation and feasibility, economic considerations, health equity, and community-level fluoride interventions
Evidence Prioritization	Priority given to systematic reviews and meta-analyses, government evidence syntheses, comparative policy analyses, jurisdictional case studies, and position statements issued by public health organizations
Application to Policy Analysis	Evidence synthesis and evaluation were guided by Bardach's policy analysis framework. Consistent with Bardach's ‘select criteria’ step, policy options were evaluated according to health impact, equity, economic impact, safety, and implementation feasibility.

Consistent with Bardach's ‘select criteria’ step, five evaluation domains were selected because they reflect core decision-making considerations commonly used in public health policy analysis and oral health systems planning. Specifically, the analysis evaluates health impact, equity, economic impact, safety, and implementation feasibility. This analysis reveals both the likely effects of each policy option and the contextual factors that influence whether policies can be effectively implemented and sustained in practice.

1. **Health impact** assesses the extent to which each policy option is likely to reduce the prevalence and severity of caries and associated treatment needs at the population level.
2. **Equity** examines how policy benefits and potential burdens are distributed across populations, particularly among groups experiencing higher disease burden or having limited access to preventive services.
3. **Economic impact** evaluates program costs, potential savings from avoided dental treatment, and broader economic consequences associated with untreated oral disease.
4. **Safety** considers the scientific evidence regarding fluoride exposure at recommended levels and the regulatory systems used to monitor drinking water quality.
5. **Implementation feasibility** assesses whether existing infrastructure, governance systems, and institutional capacity can support effective and sustained policy implementation.

## Health impact analysis

### Policy question: how does CWF influence population-level caries burden and treatment needs?

Fluoride reduces caries risk by promoting remineralization of early enamel lesions and slowing progression to cavity formation. Population-level fluoride exposure policies are intended to reduce the incidence and severity of caries across communities. Because fluoridation is delivered through public water systems, exposure can reach broad populations independent of individual utilization of dental care or preventive services, although equity impacts may vary depending on infrastructure coverage and access (19).

Evaluations of fluoridation policies must also consider broader patterns of fluoride exposure. In many countries, fluoride toothpaste is widely used, and professional topical fluoride applications are provided in clinical settings. These interventions contribute to caries prevention and may reduce baseline disease risk compared with earlier decades when topical fluoride exposure was less common. However, unlike clinical preventive services that depend on individual access to care, fluoridation provides population-level exposure independent of health service utilization.

Systematic reviews of CWF have reported lower caries prevalence in fluoridated communities compared with non-fluoridated areas (6). However, contemporary evidence suggests that the incremental benefit of fluoridation may be smaller in populations with widespread fluoride toothpaste use than in historical settings (6). Evidence from jurisdictions that discontinued fluoridation provides additional insight into potential population effects. Studies comparing Calgary, Canada, where fluoridation ended in 2011, with nearby Edmonton, where fluoridation continued, reported higher caries prevalence among children in Calgary (20). Policy analyses of this case further indicate that the decision to terminate fluoridation was shaped by political dynamics and public discourse rather than scientific evidence alone (21). These findings suggest that the discontinuation of water fluoridation may be associated with increased disease burden at the population level (22).

Reductions in disease prevalence can also influence treatment needs. Severe untreated caries may require complex restorative procedures or hospital-based dental treatment, particularly among young children requiring care under general anesthesia (23). Prevention strategies that reduce disease incidence can, therefore, decrease demand for resource-intensive treatment.

Overall, evidence indicates that CWF can reduce caries prevalence and treatment needs at the population level (3). The magnitude of these effects may vary depending on baseline disease levels, fluoride exposure from other sources, and patterns of access to preventive dental care. For these reasons, CWF is best understood as one component of broader prevention strategies aimed at reducing dental disease across populations.

## Equity impact assessment

### Policy question: how might CWF influence disparities in caries and access to prevention across different populations?

Caries is unevenly distributed across populations and strongly influenced by social and structural drivers of health. Global oral health policy reports consistently show higher disease burden among populations experiencing socioeconomic disadvantage. Across many health systems, lower-income populations and those with limited access to preventive dental services experience higher levels of untreated disease, reflecting barriers such as cost, transportation, and provider availability.

Children from disadvantaged households are particularly affected by these disparities (24, 25). Early childhood caries is more common among children living in poverty, and untreated disease may progress to conditions requiring extensive restorative procedures or hospital-based dental treatment (26).

Population-level prevention strategies are often emphasized in global oral health policy because they can reach individuals regardless of access to clinical services. CWF provides fluoride exposure through drinking water systems and may reduce disparities in preventive exposure across populations when access to centralized water systems is widespread (3).

However, the impact on equity depends on infrastructure and access to care. The magnitude of benefit may vary depending on infrastructure, patterns of water access, and other sources of fluoride exposure (27).

From an equity perspective, fluoridation policies may be understood within a proportionate universalism framework, in which population-wide interventions can provide broad benefits while potentially yielding greater absolute gains among populations with higher baseline disease burden. However, equity effects depend on whether disadvantaged populations are effectively reached. Communities relying on private wells, decentralized water systems, or limited public infrastructure may experience different levels of direct exposure to fluoridated water, although some benefits may still accrue through broader community and environmental pathways. Prevention strategies that rely primarily on clinical services may risk widening disparities when access to preventive dental care is uneven.

## Economic impact analysis

### Policy question: what are the economic costs and potential savings associated with implementing or maintaining CWF?

Untreated dental disease generates substantial economic losses: global estimates of $710 billion annually in total costs ($387 billion direct treatment costs and $323 billion productivity losses, reflecting missed work and school days, reduced workforce participation, and broader economic disruption) (10).

CWF operates through existing drinking water infrastructure, resulting in relatively low per-person costs, particularly in larger municipal systems where costs are distributed across many users. However, implementation and maintenance costs may be proportionally higher in smaller or decentralized water systems, where infrastructure and operational capacity are limited. Economic evaluations have reported that fluoridation can reduce dental treatment needs by lowering the incidence of caries and reducing spending on restorative procedures such as fillings and extractions (28). The US Centers for Disease Control and Prevention has identified fluoridation as a cost-saving public health intervention in many U.S. communities, although economic outcomes may vary across jurisdictions depending on infrastructure, population size, and baseline disease burden (29–31). Research estimates that residents of fluoridated communities in the U.S. saved $32.19 per capita because the rate of dental caries was lower in such communities (29).

## Safety and risk assessment

### Policy question: what evidence exists regarding the safety of CWF at recommended fluoride concentrations?

Public health authorities have established recommended fluoride concentrations to optimize caries prevention while limiting the likelihood of dental fluorosis. The United States Public Health Service recommends a concentration of 0.7 milligrams of fluoride per liter for CWF (32). International guidance from the World Health Organization similarly recognizes that low levels of fluoride exposure can contribute to caries prevention while higher concentrations may increase the risk of fluorosis.

Public water systems routinely monitor fluoride concentrations and adjust dosing to maintain recommended levels. Extensive reviews by public health authorities have concluded that CWF at recommended concentrations is a safe and effective strategy for preventing caries (33–36).

Safety assessments often examine dental fluorosis, a change in enamel appearance that can occur during tooth development. When fluoridation levels are maintained within recommended ranges, fluorosis is generally mild and considered cosmetic rather than a health risk (37). Severe fluorosis and skeletal fluorosis occur at fluoride concentrations substantially higher than those used in regulated CWF programs.

Some studies examining potential systemic or neurodevelopmental effects analyzed fluoride exposures that were usually substantially higher than those encountered in controlled CWF systems. At the same time, scientific and policy discussions continue regarding total fluoride exposure from multiple sources and the interpretation of emerging evidence related to neurodevelopmental effects. Current public health guidance in several countries continues to support CWF at recommended concentrations while emphasizing ongoing monitoring and evidence review.

For areas that do not engage in CWF, policymakers should be aware of the natural, pre-existing fluoride concentrations in drinking water. About 180 million people, roughly 2 percent of the global population, live in communities whose primary water sources exceed the World Health Organization's recommended fluoride limit, which is 1.5 mg/L of water. Researchers have created a global fluoride hazard map, based on machine learning, that policymakers can consult (38). Knowledge of natural fluoride levels can enable policymakers to take appropriate action, such as defluoridation or publicly advising residents to choose a different drinking water source with a lower fluoride concentration.

## Implementation feasibility, compliance and governance

The feasibility of population-level fluoride exposure policies depends on infrastructure, institutional capacity, and regulatory authority. For policymakers, feasibility considerations include whether existing water systems can support fluoridation and which institutions are responsible for oversight and compliance with public health guidance.

CWF is most easily implemented in areas served by centralized municipal water systems where treatment processes already occur. In these settings, fluoridation can be integrated into routine water treatment operations with relatively modest technical requirements.

Implementation becomes more complex in regions with decentralized water supply systems. Rural communities, small municipalities, and areas relying on private wells or small-scale systems may lack the infrastructure needed to support centralized fluoridation programs, making population-level fluoride exposure more difficult to achieve through drinking water systems (3, 39).

Governance structures also influence feasibility. In many countries, national governments establish drinking water standards and public health guidance. At the same time, regional authorities or municipal utilities operate water systems, requiring coordination between regulatory agencies, public health institutions, and local utilities.

## Implementation considerations

For policymakers, implementation planning focuses on ensuring that fluoridation programs operate consistently and maintain public confidence.

Monitoring and regulatory oversight play an important role in maintaining appropriate fluoride concentrations. Water utilities typically conduct routine testing to verify fluoride levels and ensure compliance with drinking water regulations. In many jurisdictions, environmental or public health agencies oversee reporting systems that track fluoride concentrations and support regulatory compliance (40, 41).

Public communication is also an important component of implementation. Because drinking water policies affect entire communities, policymakers often face questions regarding safety, effectiveness, and individual choice. Transparent communication about how fluoridation helps reduce caries, the safeguards used to monitor fluoride levels, and the evidence supporting its use can support informed public dialogue and strengthen public trust (42).

Integration with broader oral health prevention strategies should also be considered. Water fluoridation programs are most effective when implemented alongside other preventive measures, such as fluoride toothpaste, fluoride varnish, dental sealants, oral health education (15) and healthy nutrition.

## Recommendations

**Figure 1:**
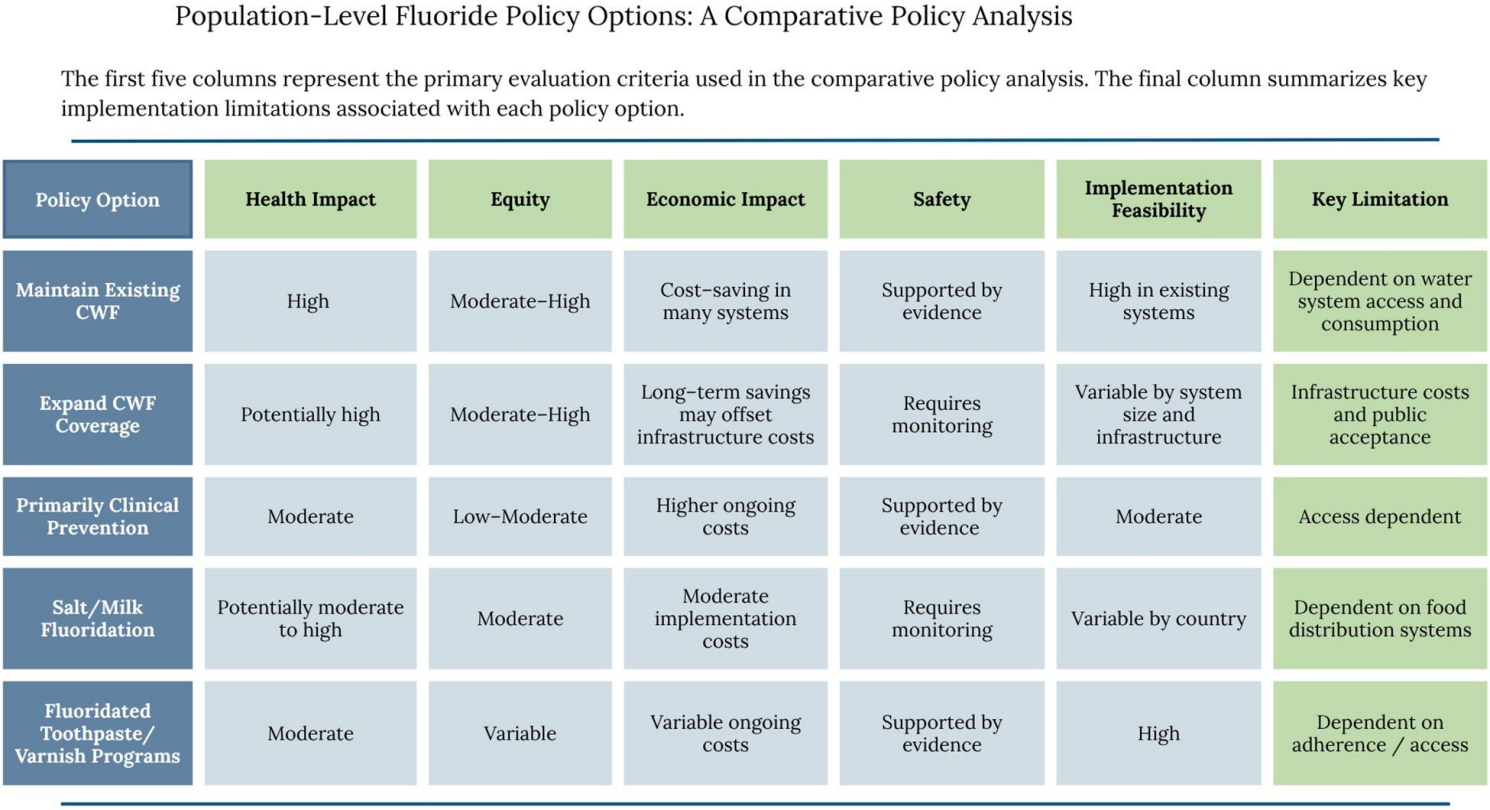
Population-level fluoride policy options: A comparative policy analysis the first five columns represent the primary evaluation criteria used in the comparative policy analysis. The final column summarizes key implementation limitations associated with each policy option.

Based on the available evidence and comparative evaluation of health impact, equity, economic impact, safety, and implementation feasibility (Figure 1), policymakers considering population-level fluoride exposure policies may consider several strategies to support effective and equitable prevention of caries.

First, governments should maintain existing CWF programs at recommended concentrations where infrastructure and regulatory capacity already exist. Maintaining programs preserves a population-level prevention strategy that can reach entire communities regardless of access to dental care.

Second, policymakers may consider expanding fluoridation coverage in jurisdictions where centralized water infrastructure makes implementation feasible. Expansion strategies should be accompanied by regulatory oversight, monitoring systems, and transparent public communication to maintain safe fluoride concentrations and support public trust. In resource-constrained settings where CWF may be less feasible, policymakers should consider alternative population-level fluoride delivery strategies, such as greater reliance on other preventive approaches tailored to local infrastructure and governance systems.

Third, governments should strengthen surveillance systems that monitor oral health outcomes and disparities across populations. Tracking indicators such as caries prevalence, untreated disease, and treatment utilization can help policymakers evaluate whether prevention strategies are achieving their intended public health impact.

Fourth, fluoridation policies should be integrated with broader oral health prevention strategies, including access to preventive dental services, fluoride toothpaste use, and community-based prevention programs. Research in oral health systems and medical–dental integration highlights the importance of coordinated prevention strategies that operate across health care and public health systems.

Fifth, policymakers should support initiatives that raise the public's oral health literacy. Even population health measures can be less effective, for example, if people are not actually drinking fluoridated tap water or adding fluoridated salt to prepared foods. These initiatives can encourage people to use these sources of fluoride, as well as instill a broader knowledge about the important roles played by dietary choices, toothbrushing, and other factors.

Sixth, policymakers should support continued research and transparent public reporting on fluoride exposure and oral health outcomes. Ongoing evaluation can help governments assess program effectiveness, respond to emerging scientific evidence, and maintain public confidence in prevention policies.

Finally, policymakers should review the regulatory framework in their geographical jurisdictions to determine whether water standards or rules are consistently applied, regardless of whether a water company is publicly or privately owned.
